# Trans-Epithelial Immune Cell Transfer during Suckling Modulates Delayed-Type Hypersensitivity in Recipients as a Function of Gender

**DOI:** 10.1371/journal.pone.0003562

**Published:** 2008-10-29

**Authors:** Lisa J. Ma, Barbara Walter, Ariel DeGuzman, H. Konrad Muller, Ameae M. Walker

**Affiliations:** 1 Division of Biomedical Sciences, University of California Riverside, Riverside, California, United States of America; 2 Institute for Integrative Genome Biology, University of California Riverside, Riverside, California, United States of America; 3 Discipline of Pathology, University of Tasmania, Hobart, Australia; Centre de Recherche Public-Santé, Luxembourg

## Abstract

**Introduction:**

Breast feeding has long term effects on the developing immune system which outlive passive immunization of the neonate. We have investigated the transfer of milk immune cells and examined the result of transfer once the recipients were adult.

**Methods:**

Non-transgenic mouse pups were foster-nursed by green fluorescent protein (GFP) transgenic dams for 3 weeks and the fate of GFP+ cells was followed by FACS analysis, immunohistochemistry and RT-PCR for GFP and appropriate immune cell markers. Pups suckled by non-transgenic dams served as controls.

**Results:**

Despite a preponderance of B cells and macrophages in the stomach contents of the pups, most cells undergoing trans-epithelial migration derived from the 3–4% of milk cells positive for T lymphocyte markers. These cells homed to the spleen and thymus, with maximal accumulation at 3–4 weeks. By sensitizing dams with an antigen which elicits a T cell-mediated delayed-type-hypersensitivity (DTH) response, we determined that nursing by a sensitized dam (compared to a non-sensitized dam) amplified a subsequent DTH response in females and yet suppressed one in males.

**Discussion:**

These results suggest that clinical evaluation weighing the pros and cons of nursing male versus female children by mothers with genetically-linked hypersensitivity diseases, such as celiac disease and eczema, or those in regions of the world with endemic DTH-eliciting diseases, such as tuberculosis, may be warranted.

## Introduction

Breast milk is a complex biological fluid providing essential nutrients for the development of newborns [1]. Beyond this however, milk contains hormones, growth factors, cytokines, sloughed mammary epithelial cells, antibodies, and viable immune cells [1], [2]. The significant benefits of breast milk are evident through a reduced risk of infection, asthma, and development of breast cancer in breast fed offspring [3]–[5]. Additionally, breast milk has been shown to benefit neurodevelopment [6].

The role of breast milk immunoglobulins in the passive transfer of immunity is well accepted and immunoglobulins from the milk of many animal species have been shown to be transported across neonatal intestinal epithelium into the circulation [7]. However, the immuno-modulatory effects of breast milk continue after the termination of breast feeding and beyond the life span of immunoglobulins in the circulation, even continuing into adulthood. For example, those breastfed as infants who subsequently received a maternal donor renal transplant, show significantly better graft survival rates compared to those not breastfed as infants [8]. Similarly, rats foster-nursed by an allogeneic dam show delayed rejection of skin grafts taken from the foster mother [9].

T cell function is poorly developed at birth, demonstrated both by an infant's poor ability to reject tissue grafts, and lower proliferation of T cells when challenged with a mitogen [10]. This environment would allow the transfer of maternal T cells, which are long-lived and could function within the offspring as transferred, or which could participate in the thymic selection process thereby influencing T cell-regulated function throughout life. Several laboratories have investigated the possible transfer of immune cells *via* the milk [11]–[16]. However, these studies examined short term transfer from concentrated cell preparations and/or did not examine which cell types were transferred, or the physiological result of transfer. In the current study, we have examined the transfer of immune cells during normal suckling.

## Methods

### Mice

A breeder pair of C57BL/6 (B6) mice that express a transgene coding for GFP under the control of the human ubiquitin C promoter (UBI-GFP/BL6GFP), which will be referred to as GFPtg, and a breeder pair of control B6 mice were obtained from the Jackson Laboratories (Bar Harbor, MD, USA). The GFPtg mice express GFP in all tissues, with B and T cells expressing higher levels. In addition, levels of GFP expression are uniform from cell to cell within a particular lineage, changing little during development [17]. Animals were housed in cages with controlled temperature and humidity and alternating 12-h light and dark cycles. The facilities were normal, non-SPF (specific pathogen-free), non-barrier facilities approved by the Association for the Assessment and Accreditation of Laboratory Animal Care International. Animals were used in accordance with current United States Department of Agriculture, Department of Health and Human Services, and National Institutes of Health Regulations. Commercial, non-sterile diet and water were given *ad libitum*.

### Fostering of Pups

Mice were coordinately mated and wildtype B6 pups were fostered by GFPtg dams. The snouts of the dams were painted with vanilla extract prior to exchanging pups to prevent pup rejection. Organs from fostered pups were collected at weeks 1, 2, and 3 of nursing and one week post weaning (week 4) for RT-PCR, immunofluorescent staining, and flow cytometry. Additionally, fostered pups were allowed to age to 4 months and were themselves mated with each other to examine lactating mammary glands from fostered females as well as small intestines from the resulting pups.

### RNA Extraction

Organs (spleen, small intestine, thymus, liver, large intestine, mesenteric lymph nodes, ileum with Peyer's patches, mammary glands, and lung) were collected from fostered pups at 1, 2, 3, and 4 weeks, and 4 months of age and frozen in liquid nitrogen. For intestinal samples, the lumen was thoroughly flushed with Dulbecco's PBS (DPBS) prior to freezing in liquid nitrogen to ensure removal of any unincorporated maternal cells. Samples were pulverized using a mortar and pestle in liquid nitrogen. Total RNA was then isolated from the samples using Tri Reagent (Sigma; St. Louis, MO, USA).

### RT-PCR Analysis

RNA concentrations were determined spectrophotometrically and equal amounts of total RNA (5 µg) were reverse transcribed using Oligo(dT)_12–18_ Primer (Invitrogen; Carlsbad, CA, USA) and M-MLV Reverse Transcriptase (Invitrogen) for 1 hour at 37°C. Primers for GFP, Oct-4, β-Casein, or GAPDH were used for PCR amplification using the AccuPrime Taq DNA Polymerase System (Invitrogen). Thirty cycles of PCR were used for GFP using the primers 5′ GCC-GAC-AAG-CAG-AAG-AAC-G 3′ (forward) and 5′ TCC-AGC-AGG-ACC-ATG-TGA-T 3′ (reverse), for Oct-4 using the primers 5′ GGG-ATG-GCA-TAC-TGT-GGA 3′ (forward) and 5′ AGC-TTG-GCA-AAC-TGT-TCT-AG 3′ (reverse), and for β-Casein using the primers 5′ ATC-CTC-GCC-TGC-CTT-GT 3′ (forward) and 5′ TGT-GGG-ACG-GGA-TTG-C 3′ (reverse) and 26 cycles for GAPDH using the primers 5′ CCT-TCC-GTG-TTC-CTA-CCC 3′ (forward) and 5′ GGT-CCA-GGG-TTT-CTT-ACT-CC 3′(reverse) at 94°C for 30 sec, 55°C (54°C for β-casein) for 30 sec, 68°C for 30 sec, followed by extension at 72°C for 7 min. Amplification products were separated on a 2% agarose gel, stained with ethidium bromide, and photographed. Each experiment was repeated at least 3 times, from at least 3 different fosters, and each repetition gave similar results.

### Immunofluorescent Staining

Organs were fixed in 4% formaldehyde in DPBS for 2 h at room temperature. Organs were then transferred into 30% sucrose in DPBS and kept at 4°C overnight before freezing in Tissue Tek OCT compound (Sakura Finetek USA, Inc., Torrance, CA, USA). Twenty micron sections were made and post-fixed in acetone and blocked with 5% FBS, rabbit IgG (1∶1000 dilution; Sigma), and mouse IgG, Fc Fragment (1∶1000 dilution; Jackson ImmunoResearch Laboratories Inc.; Westgrove, PA, USA) in DPBS. For CD4, CD8, B220, and F4/80 staining, 5% rat serum was also added to the blocking solution. For macrophage staining using tomato lectin, 0.1% Triton-X 100 was added to the blocking solution. Sections were then stained with Alexa-conjugated (1∶1000 dilution; Molecular Probes; Eugene, OR, USA) rabbit anti-GFP, and purified rat anti-mouse CD4 monoclonal antibody (1∶50 dilution; Clone H129.19; BD Pharmingen; San Diego, CA, USA), purified rat anti-mouse CD8a monoclonal antibody (1∶50 dilution; Clone 53-6.7; BD Pharmingen), purified hamster anti-mouse TCR γδ monoclonal antibody (1∶10 dilution; Clone GL3; BD Pharmingen), purified rat anti-mouse CD45R/B220 monoclonal antibody (1∶50 dilution; Clone RA3-6B2; BD Pharmingen), biotinylated-lycopersicon esculentum (tomato) lectin (1∶100 dilution; Vector Laboratories; Burlingame, CA, USA), or biotin anti-mouse F4/80 antigen antibody (1∶800 dilution; eBioscience; San Diego, CA, USA). For the purified hamster anti-mouse TCR γδ monoclonal antibody a secondary biotin anti-hamster cocktail (1∶100 dilution; BD Pharmingen) was used. For the purified rat anti-mouse CD45R/B220, CD8a, and CD4 antibodies, secondary biotin polyclonal anti-rat Igs (1∶50 dilution; BD Pharmingen) were used. The antibodies were visualized using Streptavidin-conjugated fluorophores (1∶500 dilution; BD Pharmingen or Molecular Probes). Slides were mounted with ProLong Antifade (Molecular Probes) and analyzed using the Pathway HT Confocal Microscope (BD Biosciences; Rockville, MD, USA). At least 3 different fostering experiments were performed and immunofluorescent staining of slides was conducted on tissue sections from 6 different animals. For each animal, two 20 µm sections were examined and 6 fields approximated as representative of the whole section, were visualized by confocal microscopy at 1000× magnification. Positively stained cells per field were counted and percentages were calculated.

### Flow Cytometry

Coagulated milk from GFPtg dams was obtained from the stomach of fostered pups. This was then pressed through a 70 µm cell strainer (BD Biosciences; Bedford, MA, USA). Cells were washed with DPBS twice to remove milk solids and lipids. Spleen and thymus were made into a single cell suspension by pressing the organ through a 70 µm cell strainer (BD Biosciences). For spleens, red blood cells were lysed using red blood cell lysing buffer (Sigma). Cells were counted and Fc receptors were blocked using mouse IgG, Fc Fragment (1 µg/100 µL; Jackson ImmunoResearch Laboratories Inc.; Westgrove, PA, USA). Cells were then surface stained with Allophycocyanin (APC)-conjugated rat anti-mouse CD8a monoclonal antibody (0.125 µg/50 µL; Clone 53-6.7; eBioscience), Alexa Fluor 700-conjugated rat anti-mouse CD4 monoclonal antibody (0.125 µg/50 µL; Clone RM4-5; eBioscience) and Phycoerythrin (PE)-conjugated hamster anti-mouse γδ T cell receptor monoclonal antibody (0.25 µg/50 µL; Clone GL3; BD Pharmingen). Additionally, milk cells were stained with Pacific Blue-conjugated rat anti-mouse B220 antibody (0.5 µg/50 µL; Clone RA3-6B2; eBioscience), APC-conjugated hamster anti-mouse CD3e monoclonal antibody (0.125 µg/50 µL; Clone 145-2C11; BD Pharmingen), and Biotin-conjugated rat anti-mouse F4/80 antibody (0.125 µg/50 µL; Clone BM8; eBioscience). The secondary antibody Streptavidin-PE (0.125 µg/50 µL; BD Pharmingen) was used for F4/80 staining. Cells were stained intracellularly with anti-GFP, rabbit IgG fraction, Alexa Fluor 488 antibody (0.125 µg/50 µL; Molecular Probes) and Pacific Blue rat anti-mouse/rat Forkhead box P3 (Foxp3; 0.5 µg/50 µL; Clone FJK-16s; eBioscience). Prior to intracellular staining, cells were first treated with the Cytofix/Cytoperm Kit (BD Pharmingen) as suggested by the manufacturer. Cells were fixed in 1% paraformaldehyde and were washed and resuspended in FACS buffer (5% FBS in PBS) prior to analysis. Cells were analyzed using the FACSAria Cell Sorting System (BD Biosciences) and FACS Diva Software (BD Biosciences). Wildtype B6 and GFPtg control spleens were used as compensation controls. Cells from wildtype B6 organs were used as background staining controls and this background fluorescence was subtracted from all samples. Staining results for spleen and thymus presented are the average of at least 8 different samples from at least 4 different fosters. In each case, >30,000 events were recorded. Staining of milk cells is the average of 11 different samples.

### Effect of maternal sensitization on foster pup Delayed Type Hypersensitivity

Female GFPtg mice were sensitized with an intradermal injection of fixed *Candida albicans* into both flanks at least 5 days prior to being coordinately mated. B6 pups were then foster nursed by either sensitized or non-sensitized GFPtg dams. When the foster nursed pups reached the age of 8 weeks, they were challenged with *Candida albicans* protein antigen (Alercheck, Inc., Portland, ME, USA) in the footpad. In a second set of experiments conducted with the same dams (3 months post-sensitization at time of fostering), 8 week old foster-nursed pups (6–10 per group, each group derived from 3 different dams) were sensitized with an intradermal injection of fixed *Candida albicans* into both flanks and then received a second injection (challenge) 7 days later with *Candida albicans* protein antigen in the footpad. Footpads were measured prior to the second exposure and 24 hours post-challenge (peak footpad swelling) using a micrometer (Mitutoyo, Tokyo, Japan).

### Statistical analyses

For delayed type hypersensitivity (DTH) experiments, statistical significance was determined by the Mann Whitney test. For all other experiments, statistical significance was determined using the two-tailed Student's *t* test with corrections for multiple comparisons against a single control group, where appropriate. Data are expressed as means±SEM and p<0.05 was considered statistically significant.

## Results

### Cells in pup stomach

**Figure 1:**
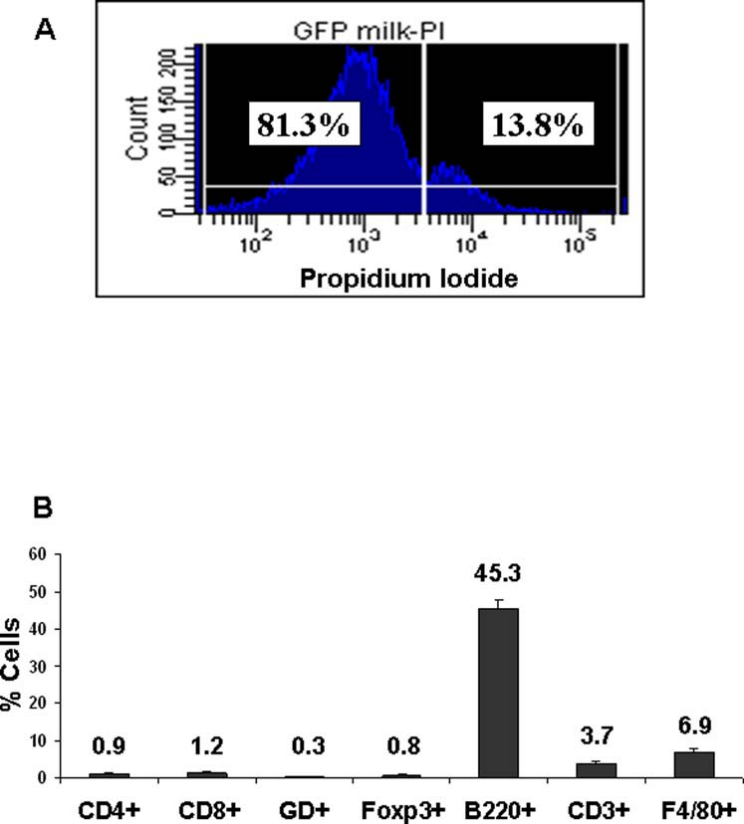
Viability and type of immune cells present in whey clot at week 2. A) Propidium Iodide staining and analysis by flow cytometry of cells isolated from the whey clot from the stomach of a pup at 2 weeks. B) percentage of milk CD4+, CD8+, γδ TCR+ (GD), Foxp3+, B220+, CD3+, and F4/80+ cells found within the clot, as determined by flow cytometric analysis.

To determine the likelihood of cell transfer from mother to pup *via* the milk under normal conditions of nursing, we first determined whether the GFP+ milk cells were viable after extraction from the stomach of the pups. Although we expected viable cells at the 1 week time point, we were surprised by the fact that greater than 80% of the cells in the milk were viable at 2 weeks. Figure 1A shows an example from 2 week old pups. Viability was assessed by the exclusion of propidium iodide, as determined by flow cytometric analysis. We next determined the proportions of cells in the milk: Analysis by flow cytometry demonstrated that 45.3±2.7% of the milk cells were B220+ (B cell marker), 6.9±0.9% F4/80+ (macrophage marker), 3.7±0.9% CD3+ (T cell marker), 1.2±0.4% CD8+ (T cell subset marker), 0.9±0.3% CD4+ (T cell subset marker), 0.8±0.2% Foxp3+ (regulatory T cell marker), and 0.3±0.03% γδ TCR+ (T cell subset marker) (Figure 1B). Remaining cells were predominantly sloughed mammary epithelium.

### Cells crossing the intestinal epithelium

**Figure 2:**
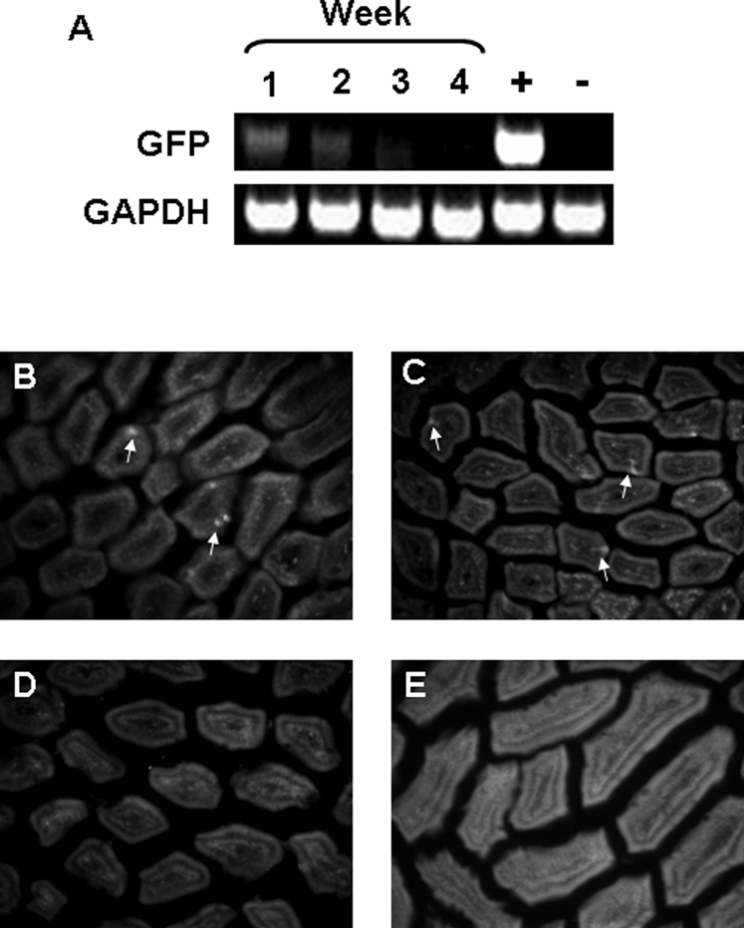
GFP+ cells cross the epithelium of the small intestine at 1 and 2 weeks of nursing. A, RT-PCR for GFP at 1, 2, 3, and 4 week time points of flushed small intestine of pups foster-nursed by GFPtg dams. The negative control used was small intestine from a non-GFP nursed B6 mouse and the positive control used was small intestine from a GFPtg mouse. B–E, sections of small intestine at weeks 1–4, respectively. Staining of GFP+ cells was enhanced with an anti-GFP antibody.

**Figure 3:**
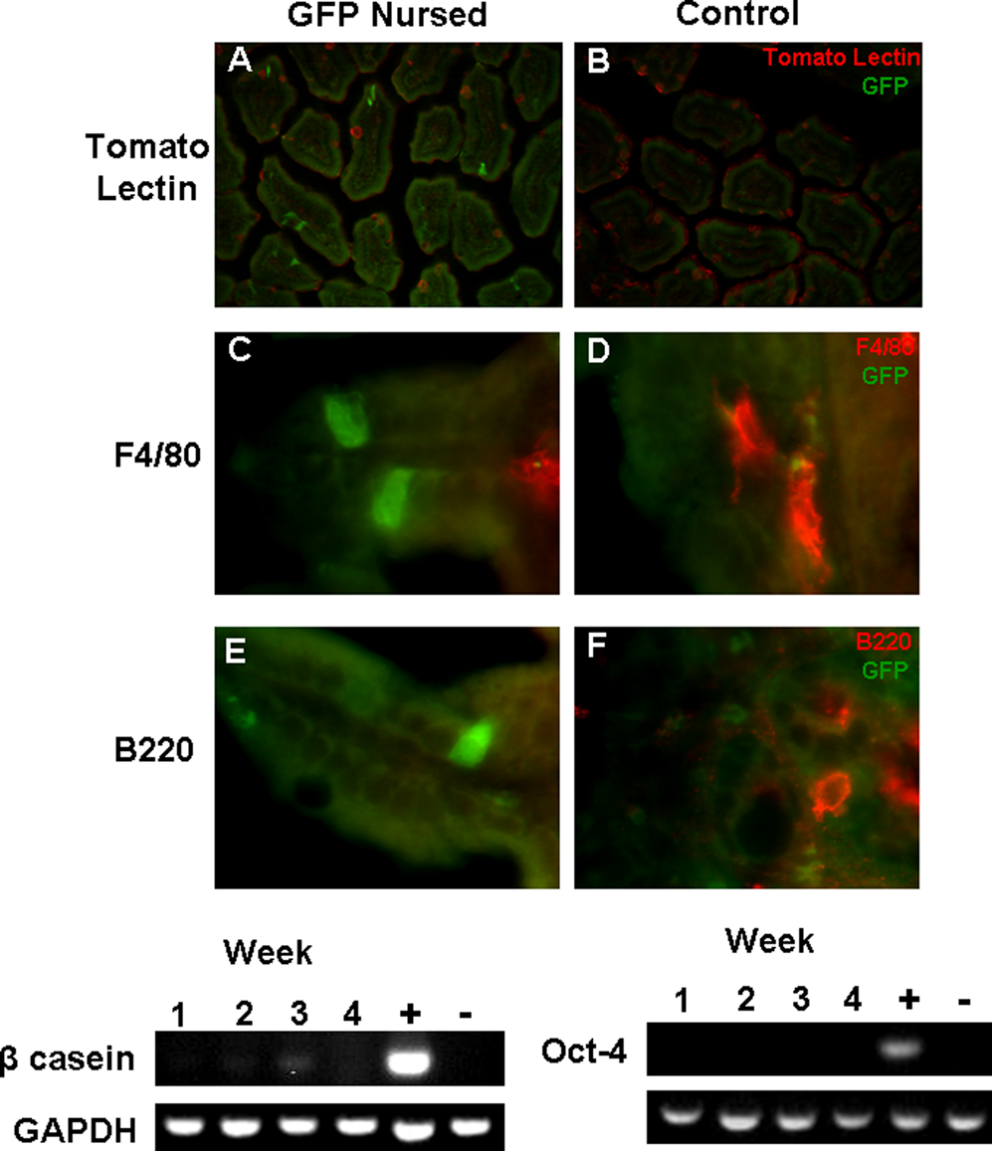
GFP+ cells crossing the epithelium of the small intestine are not macrophages, B cells, mammary epithelial cells or stem cells. Small intestine of a B6 pup nursed by a GFPtg dam stained with anti-GFP antibody and tomato lectin (A), anti-F4/80 antibody (C), anti-B220 antibody (E). Small intestine of a non-GFPtg nursed B6 control mouse stained with anti-GFP antibody and tomato lectin (B), anti-F4/80 antibody (D), or anti-B220 antibody (F). For panels A and B, anti-GFP staining is pseudo-colored green and tomato lectin is pseudo-colored red. For F4/80 and B220 staining, anti-GFP staining is in green and F4/80 and B220 is in red. Tomato lectin staining is at 200× magnification and F4/80 and B220 staining is at 1000× magnification. G, RT-PCR for β-casein at weekly time points of small intestine of pups foster-nursed by GFPtg dams. Lactating mammary gland was used as the positive control. H, similar RT-PCR for Oct-4 at weekly time points. Testis was used as the positive control.

**Figure 4:**
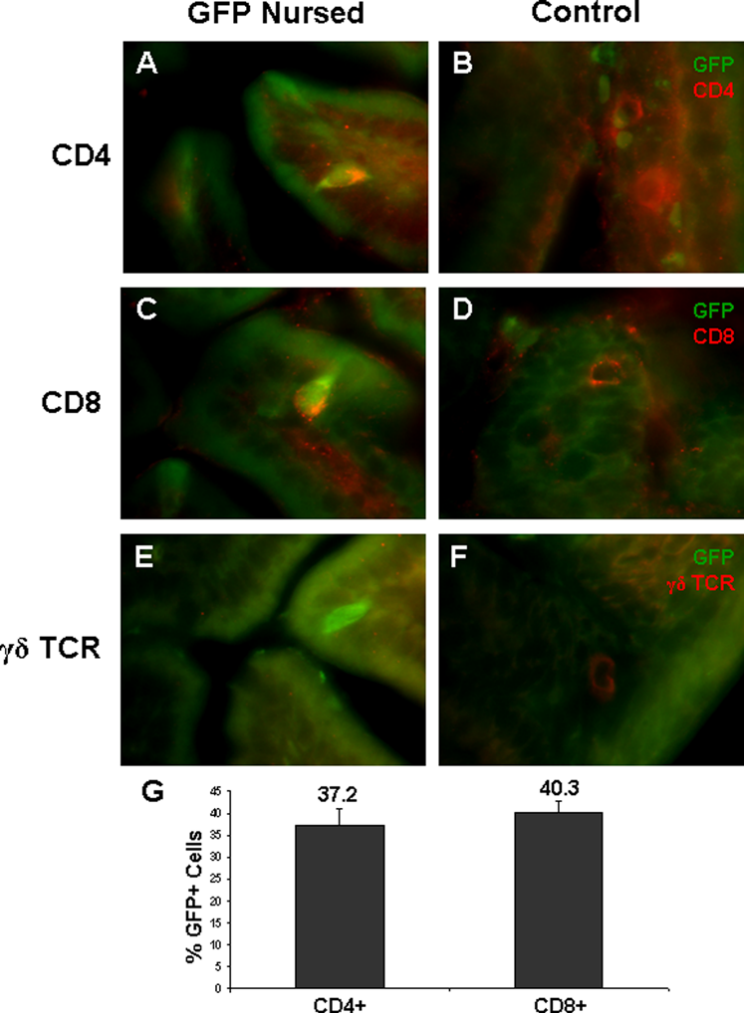
Cells crossing the epithelium of the small intestine are CD4+ and CD8+ T cells. A, small intestine of B6 pup nursed by a GFPtg dam and B, non-GFPtg nursed B6 control stained with anti-GFP antibody (green) and anti-CD4 antibody (red); C, small intestine of B6 pup nursed by a GFPtg dam and D, non-GFPtg nursed B6 control stained with anti-GFP antibody (green) and anti-CD8 antibody (red); E, Small intestine of B6 pup nursed by a GFPtg dam and F, non-GFPtg nursed B6 control stained with anti-GFP antibody (green) and anti-γδ TCR antibody (red); G, Quantification of cell types traversing the small intestine at weeks 1 and 2 (combined), as determined by immunofluorescent staining for GFP and either CD4 or CD8.

We then examined whether the GFP+ cells in milk traversed the small intestinal epithelium of the pup. Results were considered positive if at least two out of three methods utilized (RT-PCR, flow cytometry, or immunofluorescence microscopy) were conclusive. GFP+ cells were found incorporated into the epithelium of the small intestine in recipient pups continually foster-nursed by GFPtg dams at 1 and 2 weeks of age, as determined by both RT-PCR and immunofluorescent staining (Figure 2A-C). The number incorporated at week 2 was less than at week 1, as assessed by both techniques. The GFP+ cells were no longer detected in the intestinal epithelium at weeks 3 and 4 (Figure 2A, 2D, and 2E) despite continued nursing to week 3. GFP fluorescence was not as bright as anticipated and so anti-GFP was used to amplify the signal. The GFP+ cells can be seen to have an elongated morphology while passing through the epithelium, although this is better illustrated at the higher magnifications shown in Figures 3 and 4.

The next issue of importance was characterization of the cell types being transferred. Co-staining revealed that the GFP+ cells crossing the epithelium were not macrophages, as determined by both tomato lectin (in red, Figure 3A and B) and F4/80 staining (in red, Figure 3C and D). Tomato lectin also stains goblet cells [19] and F4/80 stains macrophage-like cells throughout additional layers of the small intestine [19], allowing staining of these cells to serve as positive controls. The GFP+ cells being transferred were also not B cells, as determined by the absence of B220 co-staining (in red, Figure 3E and F). Also, based on examination of sections of small intestine stained with hematoxylin and eosin, the cells were determined not to be granular and hence were not mature granulocytes or mast cells (data not shown). On the basis of RT-PCR for β-casein and Oct-4, the transferred cells were neither mammary epithelial cells nor stem cells, respectively (Figure 3G and H). There was a faint RT-PCR band for β-casein in the illustrated gel at 3 weeks, but this was not consistent among samples and did not correspond to when the GFP+ cells were observed traversing the epithelium.

Further immunofluorescent staining showed the transferred cells to be CD4+ (Figure 4A) and CD8+ (Figure 4C), but no definitive double labeling of the γδ TCR together with GFP was observed (Figure 4E). From the immunofluorescent staining, the proportions of each cell type being transferred could be determined: A similar percentage was observed, with 37.2±3.7% being CD4+ and 40.3±2.4% being CD8+ (Figure 4G). The identity of the remaining GFP+ cells is unclear at present.

### Destination of cells crossing the intestinal epithelium

After confirming the transfer of immune cells and determining the majority phenotype, we next sought to ascertain whether these cells migrated to other organs or regions. The liver, kidney, large intestine, mesenteric lymph nodes, Peyer's patches and lung were investigated by RT-PCR for GFP, but no evidence of the migration of GFP+ cells to these organs was observed during the four week time course examined (data not shown).

**Figure 5:**
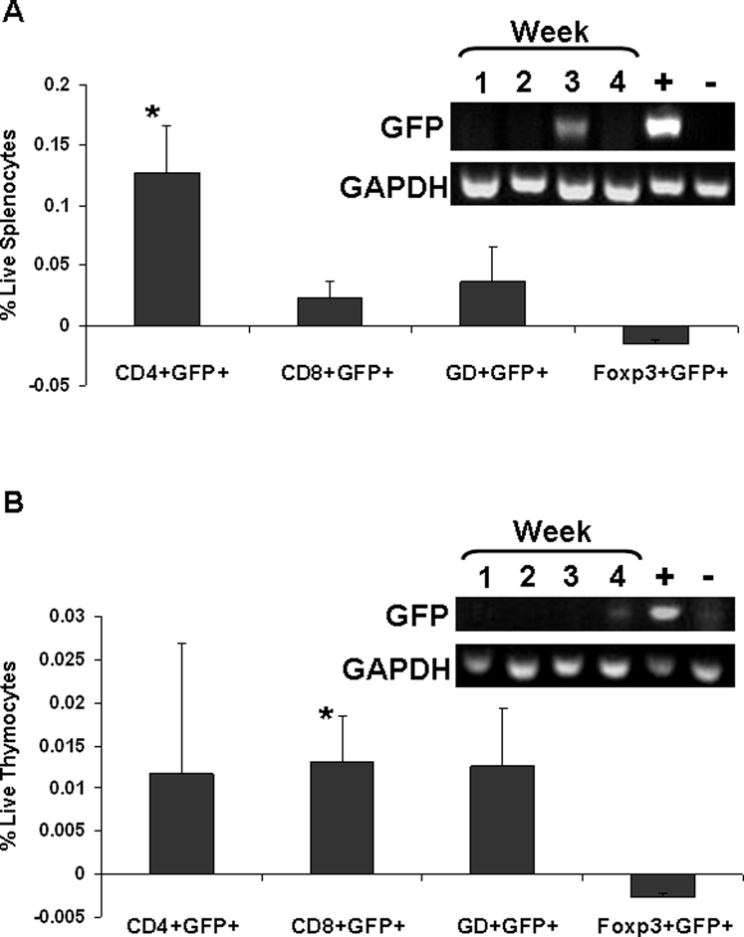
CD4+ cells migrate to the spleen and CD8+ cells migrate to the thymus. RT-PCR for GFP at weekly time points and FACS analysis of cells positive for GFP and an additional marker (CD4 or CD8 or γδ TCR (GD) or Foxp3). A, spleen; B, thymus from pups nursed by GFPtg dams. The negative controls used were spleens and thymi from non-GFPtg-nursed B6 mice, and the positive controls used were spleens and thymi from GFPtg mice. *, p<0.05 compared to non-GFPtg nursed controls.

GFP+ cells temporarily migrated to the spleen as concluded from the result of FACS analysis and RT-PCR for GFP at 3 weeks of age (Figure 5A) and the loss of these indicators by week 4. Additionally, GFP+ cells migrated to the thymus, appearing at 3 weeks of age (by immunohistochemistry- not shown), with numbers increasing by week 4 as shown by RT-PCR and FACS analysis (Figure 5B).

FACS analysis interestingly and clearly showed that CD4+ cells preferentially migrated to the spleen (Figure 5A). CD8+ cells on the other hand favored the thymus. For the thymus, this was the only cell type for which significantly different values from the controls were obtained (Figure 5B). However, with the large errors, derived from small numbers and inter-animal variation, one cannot exclude the possibility that CD4+ cells also migrate to the thymus but in a less consistent fashion. Staining for γδ TCR, Foxp3 (Figure 5A and B), or CD44 and CD127 (data not shown) could not identify any of these cells as γδ T cells, regulatory T cells, or memory T cells, respectively.

Tissues from older animals (four months old), foster-nursed by GFPtg dams, were examined to determine the longevity of transferred cells. In only one of six animals examined for this purpose, the ileum with Peyer's patches and no other tissue contained GFP+ cells. Long term survival is therefore possible, but not representative. In an additional experiment, four month old recipient mice were mated with each other. The lactating mammary glands from the females were then examined for the presence of GFP+ cells by RT-PCR. The small intestines of the pups resulting from these matings were also examined. No positive GFP RT-PCR signal was detected in either case (data not shown).

### Functional consequence of transferred cells

**Figure 6:**
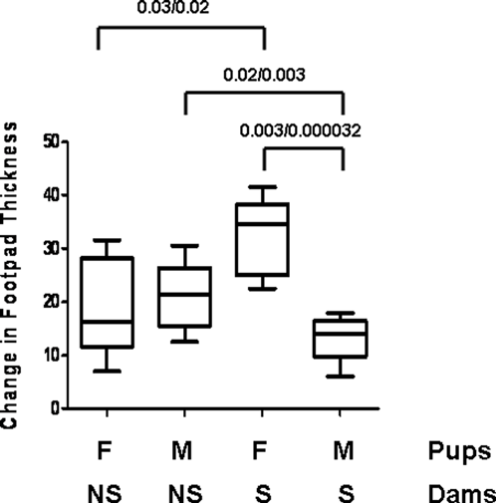
Effect of Maternal Sensitization on DTH response to *Candida* in the pups. B6 pups were foster-nursed by GFPtg dams that were either sensitized or not to *Candida*. Foster-nursed pups were sensitized with an intradermal injection of fixed *Candida albicans* into both flanks and then challenged 7 days later with *Candida albicans* protein antigen in the footpad. Footpad swelling was measured 24 hours later. Statistical analysis by both Mann Whitney (first number) and *t* test (second number) are shown. M, male; F, female; NS, non-sensitized; S, sensitized.

Since we observed the transfer of T cells, we next asked whether this transfer had any effect on a T cell-mediated response in the fostered offspring by using the DTH response to *Candida albicans*. Sensitized or non-sensitized GFPtg females were mated and then their non-transgenic foster pups were challenged with purified *Candida* antigen. There was no evidence of transferred sensitization with this protocol (data not shown). In a second set of experiments, the same dams (sensitized or not) were re-mated and at 8 weeks of age, the pups were themselves sensitized and subsequently challenged. In this experiment, there was a distinct difference in the response of pups from sensitized *versus* non-sensitized dams. If female, the DTH response of those fostered by sensitized dams was enhanced and if male, the DTH response was reduced (Figure 6). Of importance is that the males and females being compared had been nursed by the same dams. Thus, immunological information was transferred in both genders, but the outcome of that transfer was gender-specific.

## Discussion

Pioneering studies examining the transfer of immune cells *via* milk have been performed by several groups. [11]–[16]. However, other groups have been unable to show cell transfer or any benefit of foster nursing on survival or immune competence in mice or rats [20], [21]. Although these previous studies of transferred cells were exceptionally interesting, the longest time frame examined was 60 hours after ingestion of cells [11]. Additionally, previous studies usually consisted of one feeding of milk leukocytes, which were often radiolabeled and/or concentrated, and were administered using nasogastric tubes or injection directly into the digestive tract of animals starved prior to and after administration of cells [12], [13], [16], [22]. While this approach has the distinct benefit of optimizing analysis of the route of transferred cells, it is not physiological. Feeding concentrated leukocytes is also a concern since a certain amount of cell-cell fusion may occur between leukocytes and epithelial cells. In addition, this type of analysis does not test whether the concentration of leukocytes normally found in milk would result in epithelial trans-migration. In the current study, we sought to examine the transfer of maternal cells in a physiological manner. Unknown to us at the outset of the current study, one other group has examined physiological transfer using a similar approach [23]. However, in their study shorter time points were used, no transferred cell types were identified, and no immunological result of cell transfer was examined.

In our studies, pups were fostered 1–2 days after birth onto GFPtg dams where they remained until week 3 when separated for weaning. The presence of GFP+ cells in a given tissue was therefore cumulative, just as it would normally be. This allowed us to examine the phenotype of the cells in reasonable detail. Our study demonstrates that the transfer of immune cells from mother to pup *via* the milk is an event that occurs during the first two weeks of nursing since GFP+ cells were present in the epithelium of the small intestine during this time and absent at 3 weeks, despite continued nursing. Transferred cells were subsequently present in sufficient number to be observed in the spleen at 3 weeks and the thymus at 3 and 4 weeks.

During the first weeks of rodent life, digestive enzyme activities in the stomach are weak and acidity is low [24], [25], allowing for the survival of maternal milk cells. Intact stomachs of 2-day-old, well-fed mice and rats examined by Head et al. [9] showed a considerable number of intact and healthy cells dispersed in the whey clot. These cells included macrophages, lymphocytes, plasma cells, and neutrophils [9] and this information formed the basis of our initial cell type analysis. Further, macrophages harvested from the gastric contents of rats have been shown to have phagocytic activity in vitro [26]. In our study, we observed greater than 80% viability in cells extracted from the whey clot from 2 week old animals. This suggests that a reasonably hospitable environment persists for much longer than we originally anticipated and is a finding that correlates well with continued transmigration of GFP+ cells at the 2 week time point.

Our study did not show transfer of B cells or macrophages. There is some evidence in the literature for the transfer of these cells, but the experimental designs used make the physiological relevance of these observations unclear [27], [28]. It seems likely therefore that under normal physiological conditions the major function of B cells and macrophages is within the intestinal lumen of the pups.

Results in the current study establish that despite much higher numbers of B cells in the stomach contents, the vast majority of cells crossing the intestinal epithelium during physiological suckling were CD4+ and CD8+ T cells. The CD4+ and CD8+ cells made up about 80% of the cells being transferred, indicating that an as yet unidentified cell type in the milk may account for the remaining 20%. Since CD4+ and CD8+ cells collectively account for about 2% of the cells in milk, and assuming equivalent efficiency of transfer, this other cell type/s may be as little as 0.5% of those in milk and so has not been previously described. What we can deduce from the current study is that this minority population did not show characteristics of mature granulocytes, mast cells, mammary epithelial cells, or stem cells.

Since we observed the specific transfer of T cells, our goal was to examine the effect of such transfer on a T cell-mediated response. Although the half life of immunoglobulins is such that any antibody transfer during suckling would be very unlikely to have an effect at 8 weeks of age, we nevertheless chose a T cell-mediated response that does not involve antibodies, a type IV hypersensitivity response. In this kind of response, an animal is sensitized by the subcutaneous injection of a suitable antigen. The antigen is then processed by local antigen presenting cells, which prime T cells. When the animal is later challenged by injection of the same antigen at a distant site, antigen presentation again occurs locally and any primed T cells circulating through the tissue recognize the antigen and become activated. The activated cells produce soluble mediators which affect endothelial permeability and recruit an inflammatory cell infiltrate. Because there are only a few primed cells likely to be in any region of tissue at any given time, it takes some time for the inflammatory response to develop and hence the term delayed hypersensitivity response. What we hypothesized might occur was the transfer of sensitized T cells and therefore the transfer of sensitization. Contrary to our expectations, suckling did not transfer any measurable sensitization to *Candida* in the sense that the adult pups produced an inflammatory response to their first experimental exposure to the antigen. Rather, what was transferred *via* the milk was a modulation of the DTH response when the pups were both sensitized and challenged. Modulation of a response suggests regulatory T cell involvement and was the reason we examined Foxp3 expression in the GFP+ cells. However, there was no evidence of Foxp3 positivity in the GFP+ cells in the spleen or thymus. Having observed transferred cells in the thymus, we also tested the hypothesis that they were memory T cells, but they were negative for both CD127 and CD44. Memory cells are also usually long lived. The transience of the cells in the thymus alternatively suggests that they may be able to influence thymic selection and hence the development of the pup's T cell repertoire.

Even with continuous nursing, GFP+ cells were not found crossing the gastrointestinal epithelium after 2 weeks, they were not found in the spleen until 3 weeks and the thymus until 3–4 weeks. Because the phenotype of the majority was different in the two lymphoid organs, later appearance in the thymus does not seem to indicate general routing from the spleen. Most likely the different times represent accumulation maxima in each organ. The possibility of continued existence of the cells after 4 weeks was what prompted us to examine much older animals, to determine whether cells might migrate to the mammary gland in lactating animals, and to see whether cells could be seen in the tissues of a second generation. None of these analyses provided any consistent evidence of longevity beyond 3–4 weeks. An effect on immunity in the adult therefore supports an impact on development of the pup's immune system, although we cannot exclude the possibility that the cells moved to a tissue we did not examine.

The control 8 week old animals showed no difference in the DTH response between males and females. This is in contrast to older animals (12 weeks) where we [29] and others [reviewed in 30] have demonstrated a more robust response in females. Nursing by a sensitized dam did not just accelerate this gender difference because nursing also resulted in suppression of the response in the males. This suggests that whatever was transferred, was transferred to both genders, but was influenced differently by physiological context. Because the animals were not housed in a barrier facility and were therefore exposed to the normal flora and fauna of a clean animal facility, this would not be the result of simple stimulation of the dam's immune system, but rather the transfer of specific *Candida*-related information.

The gender-specific modulation raises the important question of whether this occurs in the human population. For example, is there any evidence to suggest that mothers with genetically-based type IV hypersensitivity diseases (such as eczema and celiac disease) should be advised to nurse male and not female children, and conversely whether mothers exposed to organisms which elicit a type IV hypersensitivity response (such as the tubercle bacillus) might be advised to nurse female and not male children. In this regard, an important study in the human population shows the presence of tuberculin-reactive T cells in breast fed infants of tuberculin positive mothers, but essentially none in the breast fed infants from tuberculin negative mothers [31]. Interestingly, only 8/13 infants gained tuberculin reactivity from the positive mothers, a finding that one can speculate might be explained by the other 5 being male.

The mechanisms governing gender modulation of immune responses are very complex. For adult DTH responses for example, females have a more robust response, but this does not seem to be a simple function of the major steroid hormone since both testosterone and 17β-estradiol have been shown to be suppressive [29], [32]. Unmasking the mechanism by which recipient gender influences the adult outcome of neonatal immune cell transfer will be an interesting challenge.

In conclusion, we have demonstrated trans-epithelial transfer of immune cells at one and two weeks of nursing and the subsequent appearance of these cells in the spleen and thymus. The majority of cells crossing the intestine were CD4+ and CD8+. Only CD4+ cells migrated to the spleen, while only CD8+ cells definitively migrated to the thymus. Accompanying this transfer was a long-lived modulation of a T cell-mediated response resulting in an enhanced response in females and a reduced response in males. These gender-related results may have important implications in regard to breast feeding when there is accompanying type IV hypersensitivity-related disease.

## References

[1] Ellis LA, Mastro AM, Picciano MF (1997). Do milk-borne cytokines and hormones influence neonatal immune cell function?. J Nutr.

[2] Goldman AS, Chheda S, Garofalo R, Schmalstieg FC (1996). Cytokines in human milk: properties and potential effects upon the mammary gland and the neonate.. J Mammary Gland Biol Neoplasia.

[3] Oddy WH (2001). Breastfeeding protects against illness and infection in infants and children: a review of the evidence.. Breastfeed Rev.

[4] Oddy WH (2002). The impact of breastmilk on infant and child health.. Breastfeed Rev.

[5] van Odijk J, Kull I, Borres MP, Brandtzaeg P, Edberg U (2003). Breastfeeding and allergic disease: a multidisciplinary review of the literature (1966–2001) on the mode of early feeding in infancy and its impact on later atopic manifestations.. Allergy.

[6] Agostoni C, Giovannini M (2001). Cognitive and visual development: influence of differences in breast and formula fed infants.. Nutr Health.

[7] Van de Perre P (2003). Transfer of antibody via mother's milk.. Vaccine.

[8] Campbell DA, Lorber MI, Sweeton JC, Turcotte JG, Niederhuber JE (1984). Breast feeding and maternal-donor renal allografts. Possibly the original donor-specific transfusion.. Transplantation.

[9] Head JR, Beer AE, Billingham RE (1977). Significance of the cellular component of the maternal immunologic endowment in milk.. Transplant Proc.

[10] Middleton PA, Bullock WW (1984). Ontogeny of T-cell mitogen response in Lewis rats. III. Juvenile adherent suppressor cells block adult mitogen responses.. Cell Immunol.

[11] Jain L, Vidyasagar D, Xanthou M, Ghai V, Shimada S (1989). In vivo distribution of human milk leucocytes after ingestion by newborn baboons.. Arch Dis Child.

[12] Seelig LL, Billingham RE (1981). Concerning the natural transplantation of maternal lymphocytes via milk.. Transplant Proc.

[13] Sheldrake RF, Husband AJ (1985). Intestinal uptake of intact maternal lymphocytes by neonatal rats and lambs.. Res Vet Sci.

[14] Tuboly S, Bernath S (2002). Intestinal absorption of colostral lymphoid cells in newborn animals.. Adv Exp Med Biol.

[15] Tuboly S, Bernath S, Glavits R, Kovacs A, Megyeri Z (1995). Intestinal absorption of colostral lymphocytes in newborn lambs and their role in the development of immune status.. Acta Vet Hung.

[16] Weiler IJ, Hickler W, Sprenger R (1983). Demonstration that milk cells invade the suckling neonatal mouse.. Am J Reprod Immunol.

[17] Schaefer BC, Schaefer ML, Kappler JW, Marrack P, Kedl RM (2001). Observation of antigen-dependent CD8+ T-cell/dendritic cell interactions in vivo.. Cell Immunol.

[18] Callaghan JM, Toh BH, Pettitt JM, Humphris DC, Gleeson PA (1990). Poly-N-acetyllactosamine-specific tomato lectin interacts with gastric parietal cells. Identification of a tomato-lectin binding 60–90×10(3) Mr membrane glycoprotein of tubulovesicles.. J Cell Sci.

[19] Mikkelsen HB, Mirsky R, Jessen KR, Thuneberg L (1988). Macrophage-like cells in muscularis externa of mouse small intestine: immunohistochemical localization of F4/80, M1/70, and Ia-antigen.. Cell Tissue Res.

[20] Miller SC (1981). Failure to demonstrate morphologically the presence of colostral or milk cells in the wall of the gastrointestinal tract of the suckling neonatal mouse.. J Reprod Immunol.

[21] Silvers WK, Poole TW (1975). The influence of foster nursing on the survival and immunologic competence of mice and rats.. J Immunol.

[22] Schnorr KL, Pearson LD (1984). Intestinal absorption of maternal leucocytes by newborn lambs.. J Reprod Immunol.

[23] Zhou L, Yoshimura Y, Huang Y, Suzuki R, Yokoyama M (2000). Two independent pathways of maternal cell transmission to offspring: through placenta during pregnancy and by breast-feeding after birth.. Immunology.

[24] Boass A, Wilson TH (1963). Development of mechanisms for intestinal absorption of vitamin B12 in growing rats.. Am J Physiol.

[25] Tatematsu M, Takahashi M, Tsuda H, Hirose M, Furihata C (1975). Precocious differentiation of immature chief cells in fundic mucosa of infant rats induced by hydrocortisone.. Cell Differ.

[26] Pitt J, Barlow B, Heird WC (1977). Protection against experimental necrotizing enterocolitis by maternal milk. I. Role of milk leukocytes.. Pediatr Res.

[27] Arvola M, Gustafsson E, Svensson L, Jansson L, Holmdahl R (2000). Immunoglobulin-secreting cells of maternal origin can be detected in B cell-deficient mice.. Biol Reprod.

[28] Hughes A, Brock JH, Parrott DM, Cockburn F (1988). The interaction of infant formula with macrophages: effect on phagocytic activity, relationship to expression of class II MHC antigen and survival of orally administered macrophages in the neonatal gut.. Immunology.

[29] Ma LJ, Guzman EA, DeGuzman A, Muller HK, Walker AM (2007). Local cytokine levels associated with delayed-type hypersensitivity responses: modulation by gender, ovariectomy, and estrogen replacement.. J Endocrinol.

[30] Verthelyi D (2001). Sex hormones as immunomodulators in health and disease.. Int Immunopharmacol.

[31] Schlesinger JJ, Covelli HD (1977). Evidence for transmission of lymphocyte responses to tuberculin by breast feeding.. The Lancet Sept.

[32] Carlsten H, Holmdahl R, Tarkowski A, Nilsson L-A (1989). Oestradiol- and testosterone-mediated effects on the immune system in normal and autoimmune mice are genetically linked and inherited as dominant traits.. Immunology.

